# Supplementary material for: Integrating microbial abundance time series with fermentation dynamics of the rumen microbiome via mathematical modelling
Source: PLoS One. 2024 Mar 20;19(3):e0298930. doi: 10.1371/journal.pone.0298930 (PMC10954177; doi:10.1371/journal.pone.0298930)
Supplement: Attachment — Submitted filename: reviews.docx [file pone.0298930.s001.docx]

This manuscript aims to a correlation between microbial data and the dynamics of volatile fatty acids (VFA) production during fermentation. The present study employed state observer theory to develop a model, which was validated using data from an in vitro RUSITEC experiment and an in vivo experiment involving four cows. Overall, the manuscript is coherent and clear. Good data presentation has been shown in your manuscript. I make some comments to be considered below:

1. As mentioned in line 104,” here we propose a novel approach that uses OTU data to derive microbial functional proxies for specific process of rumen metabolism such as volatile fatty acid (VFA)” Can you explain the content of OTU data in more detail?
2. As mentioned in line 118-120，“Fermentation vessels (800 mL effective volume) were inoculated with rumen fluid and incubated with the experimental diets (80:20 forage to concentrate ratio).” Why was only a single forage concentrate ratio of 80:20 selected for the experimental diet? Shouldn't multiple ratios have been considered?
3. In Tables 1 to 7, the terms Cow1 Grass, Cow1, and Cow1 Hay have been frequently observed. Although the term Cow1 Grass has been used to differentiate them from Cow1 originating from different experiments, the abundance of these occurrences can still lead to confusion among data objects. Therefore, it is necessary to explore alternative methods for distinguishing between these two groups of subjects.
4. Line 288-289, which section does this paragraph belong to? If it is a description of the contents in the table 3, please check the format requirements.
5. The thickness of the lines in Fig 1 and Fig 2 are inconsistent. The chart presentation in a paper should adhere to standardized formatting.
