# Supplementary material for: Integrating microbial abundance time series with fermentation dynamics of the rumen microbiome via mathematical modelling
Source: PLoS One. 2024 Mar 20;19(3):e0298930. doi: 10.1371/journal.pone.0298930 (PMC10954177; doi:10.1371/journal.pone.0298930)
Supplement: Attachment — Submitted filename: 20231221_Response_Letter_RumenObserver_Munoz-Tamayo.docx [file pone.0298930.s002.docx]

**MoSAR-Systemic modelling applied to ruminants**

Université Paris-Saclay, INRAE, AgroParisTech,

91120 Palaiseau, France

Tel. : + 33 7 62 57 24 96

[Rafael.munoz-tamayo@inrae.fr](mailto:Rafael.munoz-tamayo@inrae.fr)

Palaiseau, France, December 21 ^st^ 2023

Dear Dr. Adham A. Al-Sagheer

Academic Editor PLOS ONE

We have addressed the points raised during the review process. Below are the responses to the comments. We hope you find the paper suitable for publication in PLOS ONE.

On behalf of the authors,

Rafael Muñoz-Tamayo

**Reviewer 1**

1. As mentioned in line 104,” here we propose a novel approach that uses OTU data to derive microbial functional proxies for specific process of rumen metabolism such as volatile fatty acid (VFA)” Can you explain the content of OTU data in more detail?

R. Information on OTU data is detailed in L201-211. We added a sentence for clarification: L210.

2. As mentioned in line 118-120，“Fermentation vessels (800 mL effective volume) were inoculated with rumen fluid and incubated with the  experimental diets (80:20 forage to concentrate ratio).” Why was only a single forage concentrate ratio of 80:20 selected for the experimental diet? Shouldn't multiple ratios have been considered?

R. It should be noted that the RUSITEC data is already published. We used the data as case study. It will be indeed useful to test our approach with animals fed at different diets including diets supplemented with additives impacting rumen fermentation patterns. We have added this comment on L298-300.

3. In Tables 1 to 7, the terms Cow1 Grass, Cow1, and Cow1 Hay have been frequently observed. Although the term Cow1 Grass has been used to differentiate them from Cow1 originating from different experiments, the abundance of these occurrences can still lead to confusion among data objects. Therefore, it is necessary to explore alternative methods for distinguishing between these two groups of subjects.

R. To distinguish between the *in vitro* and the *in vivo* studies, the terms were modified for tables 1, 2, 3. We used “Rumen inoculum” instead of “Cow” for the *in vitro* study.

4. Line 288-289, which section does this paragraph belong to? If it is a description of the contents in the table 3, please check the format requirements.

R. Yes, the paragraph is connected to Table 3.

5. The thickness of the lines in Fig 1 and Fig 2 are inconsistent. The chart presentation in a paper should adhere to standardized formatting.

R. We do not understand the comment about the thickness of the lines. We have enhanced the Figures using the PACE tool.

**Reviewer 2**

Reviewer 2 gave a positive assessment of our manuscript. However, for the question: “1. Is the manuscript technically sound, and do the data support the conclusions? ”She/He responded NO. We think that this is mistake and that the reviewer meant YES.

**Journal requirements**

1. Please ensure that your manuscript meets PLOS ONE's style requirements, including those for file naming. The PLOS ONE style templates can be found at

https://journals.plos.org/plosone/s/file?id=wjVg/PLOSOne_formatting_sample_main_body.pdf and

<https://journals.plos.org/plosone/s/file?id=ba62/PLOSOne_formatting_sample_title_authors_affiliations.pdf>

R. The article is now in the format of PLOS ONE.

2. We noticed you have some minor occurrence of overlapping text with the following previous publication(s), which needs to be addressed:

Integration of microbial time series into a mechanistic model of the rumen microbiome under the Rusitec condition - https://doi.org/10.1016/j.anscip.2022.07.443

In your revision ensure you cite all your sources (including your own works), and quote or rephrase any duplicated text outside the methods section. Further consideration is dependent on these concerns being addressed.

R. The reference identified is an abstract proceeding. We have included it (Davoudkhani et al. 2022) in the text: L98-101.

3. Thank you for stating the following financial disclosure:

“All authors receive funding from the MASTER project, an Innovation Action funded by the European Union’s Horizon 2020 research and innovation programme under grant agreement No 818368.”

Please state what role the funders took in the study.  If the funders had no role, please state: "The funders had no role in study design, data collection and analysis, decision to publish, or preparation of the manuscript."

If this statement is not correct you must amend it as needed.

Please include this amended Role of Funder statement in your cover letter; we will change the online submission form on your behalf.

R. The information was included in the cover letter: "The funders had no role in study design, data collection and analysis, decision to publish, or preparation of the manuscript." We deleted the section of funding in the manuscript as advice in the author’s instructions.

4. We noted in your submission details that a portion of your manuscript may have been presented or published elsewhere. [Belanche, A., Newbold, C.J., Lin, W., Rees Stevens, P., Kingston-Smith, A.H., 2017. A systems biology approach reveals differences in the dynamics of colonization and degradation of grass vs. hay by rumen microbes with minor effects of vitamin E supplementation. Front. Microbiol. 8. https://doi.org/10.3389/fmicb.2017.01456]. Please clarify whether this [conference proceeding or publication] was peer-reviewed and formally published. If this work was previously peer-reviewed and published, in the cover letter please provide the reason that this work does not constitute dual publication and should be included in the current manuscript.

R. As it can be seen, the reference of the article of Belanche et al is a peer-review published paper. The paper submitted to PLOS ONE cannot be considered as dual publication, since our objective here is focused on the modelling developments which have not been published elsewhere. The data of the article of Belanche is used as case study. I informed to the editorial office about this situation before the submission of the paper. Please find below the response by Erika Mann:

“De : plosone <plosone@plos.org>

Envoyé : lundi 11 septembre 2023 19:17

À : Rafael Munoz-Tamayo

Objet : Question criterion for publication in PLOS ONE

Dear Dr. Muñoz-Tamayo,

Thanks for your interest in submitting to PLOS ONE!

Yes, you may submit a study that uses published data as long as the results of your study have not been published elsewhere.

Based on your explanation, your article would be considered in PLOS ONE.

We look forward to receiving your submission.

Kind regards,

Erika Mann (she/her)

Peer Review Operations Manager”

5. When completing the data availability statement of the submission form, you indicated that you will make your data available on acceptance. We strongly recommend all authors decide on a data sharing plan before acceptance, as the process can be lengthy and hold up publication timelines. Please note that, though access restrictions are acceptable now, your entire data will need to be made freely accessible if your manuscript is accepted for publication. This policy applies to all data except where public deposition would breach compliance with the protocol approved by your research ethics board. If you are unable to adhere to our open data policy, please kindly revise your statement to explain your reasoning and we will seek the editor's input on an exemption. Please be assured that, once you have provided your new statement, the assessment of your exemption will not hold up the peer review process.

R. The data was already available at the first submission: L353-355.

6. Your ethics statement should only appear in the Methods section of your manuscript. If your ethics statement is written in any section besides the Methods, please move it to the Methods section and delete it from any other section. Please ensure that your ethics statement is included in your manuscript, as the ethics statement entered into the online submission form will not be published alongside your manuscript.

R. Done: L149-153.

7. Please review your reference list to ensure that it is complete and correct. If you have cited papers that have been retracted, please include the rationale for doing so in the manuscript text, or remove these references and replace them with relevant current references. Any changes to the reference list should be mentioned in the rebuttal letter that accompanies your revised manuscript. If you need to cite a retracted article, indicate the article’s retracted status in the References list and also include a citation and full reference for the retraction notice.

R. With respect to the previous item 3, we included the reference [31] of our abstract (Davoudkhani et al. 2022).

While revising your submission, please upload your figure files to the Preflight Analysis and Conversion Engine (PACE) digital diagnostic tool, <https://pacev2.apexcovantage.com/>. PACE helps ensure that figures meet PLOS requirements. To use PACE, you must first register as a user. Registration is free. Then, login and navigate to the UPLOAD tab, where you will find detailed instructions on how to use the tool. If you encounter any issues or have any questions when using PACE, please email PLOS at [figures@plos.org](mailto:figures@plos.org). Please note that Supporting Information files do not need this step.

R. Figures were enhanced using PACE.
